# What social determinants outside paid work are related to development of mental health during life? An integrative review of results from the Northern Swedish Cohort

**DOI:** 10.1186/s12889-021-12143-3

**Published:** 2021-11-30

**Authors:** Shirin Ziaei, Anne Hammarström

**Affiliations:** 1 Unit of Occupational Medicine, Institute of Environmental Medicine, Karolinska Institutet, Solnavägen 4, 113 65 Stockholm, Stockholm Sweden; 2 Department of Epidemiology and Global Health, Umea University, 901 87 Umea, Sweden

**Keywords:** Mental health, Social determinants, Outside paid work, Bronfenbrenner, Integrative review

## Abstract

**Background:**

Despite global increase in burden of mental health conditions, longitudinal studies on factors related to development of mental health are scarce. Particularly integrated understanding of how factors at each level of ecological system interact to influence mental health of individuals during their life is missing. Both work and outside work (life beyond work) spheres are two important areas in human life which can have independent effects on mental health of individuals. In this integrative review, we aimed to synthesis findings about social determinants outside paid work that are related to development of mental health during life in a 27-year prospective Swedish Cohort study by using Bronfenbrenner’s Ecological Systems Theory.

**Methods:**

The material for this paper consists of all mental health related papers within Northern Swedish Cohort. Papers related to outside paid work exposures of life circumstances were selected. An integrative review was conducted on 27 papers and deductive qualitative content analysis in relation to Bronfenbrenner ecological framework was performed to identify the main themes.

**Results:**

The results of this review showed that class structures and gender order at macro-level permeated into all other levels and finally became embodied in the individuals as symptoms of mental health during life. At the “exo-level” neighbourhood disadvantage was related to mental ill-health of individuals. The importance of parental interaction with other settings, like school, for mental health of individuals was highlighted at “meso-level”. At “micro-level” poor social relationships; social and material adversities and inequality in gender relations during adult life were related to mental ill-health.

**Conclusion:**

We found mental health of individuals to be related to both unique and common factors manifesting at different socio-ecological levels. Social structures at the macro-level namely class structures and gender order permeate all other levels and eventually become embodied in the individuals as symptoms of mental health during life. Interventions addressing gender and class related inequalities might be of importance for improving mental health of individuals during their life.

## Background

The global burden of mental health conditions has been raised during the last decade. Nowadays, mental health conditions and substance abuse is one of main factors related to years lived in disability worldwide [1, 2]. According to World Health Organization (WHO), mental health is “a state of well-being in which every individual realizes his or her own potential, can cope with the normal stresses of life, can work productively and fruitfully, and is able to make a contribution to her or his community “[3]. It is widely accepted that mental health is the result of a complex interplay between individual, social, economic and environmental factors in various stages of lives. Since the exposure, impact and vulnerability to such factors varies across the life, more comprehensive approach is needed in order to understand and develop proper mental health interventions [4]. Despite the importance, longitudinal studies on determinants of mental health are scarce [5].

Bronfenbrenner socio-ecological model on human development is a useful tool to examine multiple and interactive factors that influence individual’s mental health. Bronfenbrenner suggested that one’s psychological development is shaped by five concentric mutually influencing layers of micro-, meso-, exo-, macro- and chrono-levels moving from the most proximal to the most distal level. In the centre of Bronfenbrenner model is the individual human beings and their personal, biological and psychological characteristics. Micro-level encompasses individual’s immediate settings and close relationship such as home, school, family and friends. Meso-level refers to interaction between two or more micro-level settings in which the individual is an active participant such as interactions between family and school. The exo-level includes settings in which the individual is not an active participant but can influence him/her nonetheless, such as parental working conditions. Macro-level implies broader institutional pattern of culture and subculture such as economic, social and political systems which can influence the nature of interaction within all other levels. And finally, chrono-level reflects the changes in individual or environment over the time [6, 7]. Bronfenbrenner believed that in order to understand human development and mental health the entire ecological system surrounding the individual must be taken into consideration [8].

Both work and outside work spheres are two important areas in human life which can manifest at multiple ecological levels and play important roles in mental health. The work-related sphere reflects experiences of individuals in relation to the work-domain such as unemployment and other aspects of employment status, labour market attachment and work environment (job insecurity, job demand, job control); while the outside work sphere refers to life beyond work such as social and material conditions of living, quality and quantity of social relationships, the neighbourhood that the individuals belonged to, etc. Although there can be great interface between them [9], studies have shown that work and outside paid work domains can have independent effects on mental health of individuals [10, 11].

The independent effect of several social determinants outside paid work such as adverse life conditions, social isolation, and economic disadvantages on mental health of individuals has been highlighted previously [10] However, longitudinal studies on determinants of mental health outside paid work are rare [12] and particularly limited research with more holistic view on the importance of social determinants outside paid work for mental health of individuals is available.

Sweden is a country ranking high in income and gender equality [13]. The country is well known for its egalitarian policies and generous welfare system providing social assistance and a wide range of tax-funded, publicly available social services such as child and elderly care, long parental leave, and low cost for the use of health care [14]. However, there is evidence of consistent socioeconomic gradient in mortality in the country [15, 16]. In addition, like in other countries, there are gender differences in the prevalence of certain disorders such as musculoskeletal and mental health disorders [17, 18]. Besides, research worldwide as well as in Sweden reveal gender bias in access, counselling, assessment and treatment of some disorders such as chronic pain in primary and specialty care in the country [19–21]. Similarly, despite the great gender equality policies, inequalities in family, working life and social insurance remain between men and women [22–24].

This review aimed to identify social determinants outside paid work that are related to development of mental health from adolescence until midlife in a Swedish population. A large research program has been funded for applying Bronfenbrenner’s ecological theory of human development and life-course analyses of the development of mental health in a 27-year follow-up of the Northern Swedish Cohort (NoSCo). The review is based on the findings from that program, by using Bronfenbrenner’s Ecological Systems Theory.

## Method

In order to obtain broader and more holistic knowledge regarding outside paid work determinants of mental health over life, we have conducted an integrative review of papers from the Northern Swedish Cohort (NoSCo) by adapting the Whittemore and Knafl’s framework [25]. Integrative review is a type of review which summarizes past empirical and theoretical studies and enables inclusion of studies with diverse methodologies [25]. Integrative review has been selected as it is a useful approach to address mature topics in order to re-conceptualize the expanding and diverse literature on that topic and provide more comprehensive understanding of phenomenon of interest [26, 27].

### Context

The Northern Swedish Cohort is a 27-year longitudinal prospective study including all pupils in Luleå, a middle-sized town in north of Sweden, who attended their last year of compulsory school (ninth grade) during 1981. Of 1083 invited pupils (506 girls, 577 boys), 1080 participated in the baseline study. The participants were followed up during 1983, 1986, 1995 and 2008 at the ages of 18, 21, 30 and 43, respectively with extensive questionnaires, clinical measures and register data. The baseline questionnaire (age 16) was performed in the classrooms while the follow ups were conducted in class reunions or via postal questionnaires.

During each waves of investigation, participants completed a comprehensive questionnaire regarding their health status, health behaviours, socio-economic status and social conditions etc. [28]. Questions measuring mental health and somatic symptoms were developed to evaluate anxiety symptoms (including restlessness; concentration difficulties; worrying; palpitations or stomach problems; and anxiety or panic), depressive symptoms (including sleeping problems; poor appetite; general tiredness; feeling down and sad; dejected about the future and concentration difficulties) and functional somatic symptoms (including headache or migraine; other stomach ache than heartburn, gastritis or gastric ulcer; nausea; backache; hip pain or sciatic; breathlessness; dizziness and overstrain) among participants [29]. The mental health measurements have been evaluated and displayed adequate psychometric property with acceptable internal consistency and factorial variance over the time [29].

Additionally, several qualitative subsamples have been drawn for personal interviews. One consists of all 13 girls and 15 boys who were unemployed directly after compulsory school. This group has been repeatedly interviewed during the cohort period [28]. Further, at the age of 47, 4 women and 4 men were strategically chosen from their answers about gender equality in the couple relationship in the questionnaires with the purpose of reaching people in different situations of gender equality. They were interviewed to explore their experiences of health in relation to how gender equal their relationship was [30].

The attrition has been extremely low in NoSCo with response rate of 94.3% (of those still alive) at the latest follow up in 2008. The cohort has been shown to be a fair representative of the whole Swedish population with regards to socio-demographic factors as well as socio-economic and health status [28]. The study has been approved by reginal ethical review board in Umeå, Sweden [28].

### Search strategy

In the first step, an updated list of all mental health related publications within NoSCo was compiled. Mental health was defined as internalized symptoms or externalized behaviours including alcohol consumption. In young age, mental health difficulties are usually represented in two domains, internalized and externalized symptoms antecedents with high levels of co-morbidity, reciprocal relations and common antecedents [29]. Due to our longitudinal design from adolescent internalized and externalized symptoms, we included alcohol misuse (Heavy Episodic Drinking) as a sign of mental ill-health in adulthood as also recommended by WHO [4]. In order to be included in this review, the papers must have mental health as an outcome and contain outside paid work exposures of life circumstances. From the total of 48 mental health related papers in NoSCo, 21 paper were excluded (15 were work-related and 6 had no exposure), resulted in final sample of 27 papers.

The NoSCo participants were targeted at the age just before entering job market. Most grants to cohort analyses have focused on various aspects of the working life and thus several papers in NoSCo are related to their working and employment conditions. We aim to evaluate work-related papers in another review.

### Data extraction

To extract relevant data, the selected papers were carefully reviewed. A data extraction spreadsheet was created to record the following factors: a) context, b) exposures, c) causal mechanism (if any), d) the main findings and e) any other relevant information. The overall context was similar in all the papers reflecting a closed cohort of all school leavers, followed up to age 43.

Thereafter, qualitative deductive content analysis methodology in relation to Bronfenbrenner ecological framework [6, 7] was conducted. Bronfenbrenner’s framework was used in order to focus on multiple and interactive contextual factors that influence the development of individual’s mental health over life. Content analysis is a method of analysing written and verbal communication in a systematic way [31, 32]. The analysis can be either theory or data driven [33]. In this study we have used theory-driven analysis to be able to contextualize our findings in relation to Bronfenbrenner’s’ theory. Themes and subthemes are organized based on his theory, which is a move from theory to data or from a more abstract and general to a more concrete and specific level [32].

The key findings regarding factor b, c and d (b = exposures, c = causal mechanism (if any), d = the main findings) in the data extraction sheet were coded and categorized based on Bronfenbrenner’s description of ecological levels. Further within each level the codes were clustered based on their thematic similarities into emergent subthemes. Related subthemes were grouped into themes. Subthemes can be seen as the thread of meaning which runs through the text while themes represent the underlying meaning of the text which answer the question “how” [32].

## Results

A total of 27 papers were included in this review. Two of the papers were qualitative and the rest were quantitative studies with cohort design, and they have been published from 2011 to 2020. Table 1 displays the detail information about the included papers.

**Table 1:** Summary information of the studies included in the review

Year of publication	Authors	Study design	Exposure	Age at exposure	Outcome	Age at outcome	Level
2015	Gustafsson et al. [34]	Quantitative	Cumulative neighbourhood and individual disadvantages	16–43	Functional somatic symptoms	43	Exo-level/Micro-level
2014	Gustafsson et al. [35]	Quantitative	Neighbourhood disadvantage	16–43	Allostatic load	43	Exo-level
2017	Gustafsson et al. [36]	Quantitative	Neighbourhood disadvantage	16–43	Functional somatic symptoms	43	Exo-level
2020	Berg et al. [37]	Quantitative	Exposure to drunkenness-oriented drinking culture in school	16	Heavy episodic drinking	43	Exo-level
2015	Westerlund et al. [38]	Quantitative	Parental involvement in their offspring’s studies	16	Internalized mental health symptoms	16 to 43	Meso-level
2013	Westerlund et al. [39]	Quantitative	Parental involvement in their offspring’s studies	16	Allostatic load	43	Meso-level
2018	Wiklund et al. [40]	Qualitative	Parental interaction with destructive relationship	14	(Mental) Health experiences	33	Meso-level
2015	Landstedt et al. [41]	Quantitative	Poor relation with parents and peers	16	Internalized mental health and Functional somatic symptoms	21,30 and 43	Micro-level
2018	Berg et al. [42]	Quantitative	Poor relation with family and classmates	16	Heavy episodic drinking	21,30, 43	Micro-level
2019	Bean et al. [43]	Quantitative	Poor relation with peers	16	Depressive symptoms	43	Micro-level
2019	Nyberg et al. [44]	Quantitative	School connectiveness and family climate	16	Depressive and anxiety symptoms	43	Micro-level
2017	Almquist et al. [45]	Quantitative	Social support	30, 43	Depressive symptoms	30,43	Micro-level
2016	Landstedt et al. [46]	Quantitative	Social Support	30	Internalized mental health symptoms	43	Micro-level
2014	Jonsson et al. [47]	Quantitative	Social capital	16, 21, 30 and 43	Functional somatic symptoms	43	Micro-level
2016	Landstedt et al. [48]	Quantitative	Social capital	16,21,30, 43	Depressive symptoms	16, 21, 30, 43	Micro-level
2015	San Sebastian et al. [49]	Quantitative	Social and material adversities	16.21.30, 43	Inequality in functional somatic symptoms	16, 21, 30, 43	Micro-level
2011	Hammarstrom et al. [50]	Quantitative	Social and material adversities	16, 21, 30	Inequality in functional somatic symptoms	16, 21, 30	Micro-level
2016	Jonsson et al. [51]	Quantitative	Socio-economic status	16	Functional somatic symptoms	43	Micro-level
2016	Rajaleid et al. [52]	Quantitative	Social adversities	16	Internalized mental health symptoms	16 to 43	Micro-level
2018	Almquist et al. [53]	Quantitative	Social and material adversities	16	Self-related health	43	Micro-level
2012	Harryson et al. [54]	Quantitative	Gender inequality in domestic sphere	43	Psychological distress	43	Micro-level
2012	Hammarstrom & Phillips [55]	Quantitative	Gender inequality in domestic sphere	43	Depressive symptoms	43	Micro-level
2011	Phillips & Hammarstrom [56]	Quantitative	Perceived gender inequality in the couple relationship	43	Self-related health	43	Micro-level
2012	Mansdotter et al. [57]	Quantitative	Childhood gender experience and adulthood gender position	16, 30, 43	Depressive and anxiety symptoms	43	Micro-level
2016	Landstedt et al. [58]	Quantitative	Changes in housework over the course of adulthood	30 to 43	Functional somatic symptoms	43	Micro-level
2012	Harryson et al. [59]	Quantitative	Gender inequality in domestic sphere and couples’ relative socio-economic position	43	Psychological distress	43	Micro-level
2016	Harryson et al. [30]	Qualitative	Housework experiences and practices	47	Experiences of stress and perceived wellbeing from a gender perspective.	47	Micro-level

In general, women reported more symptoms of internalized disorders such as anxiety, depression and somatic symptoms [34, 59, 60] while alcohol misuse was higher among men [42, 61]. Measuring the symptoms of ill mental health at the age of 16, 21, 30 and 43 indicated that depressive, anxiety and somatic symptoms co-exist from the adolescence till midlife among the participants and the level of symptoms at each point was strongly associated with previous and future level of the symptoms [60].

The findings of this review were classified into broader themes and categorized according to Bronfenbrenner ecological levels (Table 2). The dimension of time (Chrono-level) has been integrated into all levels by longitudinal design of the studies.

**Table 2:** Summary of meaning units, subthemes and themes according to Bronfenbrenner ecological levels

Macro-level	Class structures Gender order		
	**Meaning unit**	**Subtheme**	**Theme**
**Exo-level**	Cumulative neighborhood disadvantage between the age of 16–43 was associated with Functional Somatic Symptoms (FSS) (in women) [34] and allostatic load (in men) at the age of 43 [35].	Cumulative neighborhood disadvantage over the life was related to later mental ill-health.	***Relation between neighborhood disadvantage and mental ill-health during life***
	Neighborhood of living across the life course explained variation in FSS in mid-adulthood with little independent contextual contribution by neighborhood environment in adolescence [36].		
	Exposure to positive attitudes towards heavy alcohol consumption at school class-level was associated with Heavy Episodic Drinking (HED) at the age of 43 [37].	Early exposure to positive attitudes towards drunkenness at class-level (as the representative of neighborhood) was related to later alcohol misuse.	
**Meso-level**	Parental involvement in their offspring’s studies at the age of 16 was associated with more favorable trajectory of Internalized mental health symptoms (IMHS) from the age of 16–43 [38].	Good parental interaction with school in early life was related to mental health over life.	***Relation between parental interaction with other settings and mental health during life***
	Parental involvement in their offspring’s studies at the age of 16 was associated with lower alosthetic load at the age 43 [39].	Good parental interaction with school in early life was related to later mental health.	
	Parental involvement in ceasing an abusive relationship in adolescent was associated with more favorable mental health in early adulthood (age 33) [40].	Parental interaction with destructive relationship in early life was related to mental health in early adulthood.	
**Micro- level**	Poor relationship with parents at the age of 16 was associated with IMHS at the age of 30 and FSS at the age of 30 and 43 [41].	Poor relationship with parents /family in early life was related to later mental ill-health and alcohol misuse.	***Relation between poor social relationships and mental ill-health during life***
	Poor relationship with family at the age of 16 was associated with an increased likelihood of HED in men (age 21/30) [42].		
	Poor relationship with peers at age 16 years was associated with depressive symptoms [43], IMHS and FSS at age 43 years [41].	Poor relationship with peers in early life was related to later mental ill-health and alcohol misuse.	
	Poorer relation with classmates at the age of 16 were associated with an increased likelihood of HED among women at the age of 30 [42].		
	Good school connectiveness at the age of 16, was associated with lower level of depressive and anxiety symptoms at the age of 43. Professional and social establishment in early adulthood appear to partially mediate the association [44].	Good school connectiveness in early life was related to later mental health.	
	Structural and functional support at the age of 30 and 43 were associated with depressive symptoms at ages 30 and 43 both in men and women [45].	Poor social support in early adulthood was related to current and later mental ill-health.	
	Poor social support at the age of 30 was associated with IMHS at the age of 43 both in men and women [46].		
	Lower level of social capital at the age of 16 was associated with FSS at the age of 43 among men [47].	Social capital in early life was related to later mental health.	
	Higher levels of youth civic engagement (as a determinant of social capital) predicted a decrease in depressive symptoms among men in early adulthood (age 21) [48].		
	Lower levels of social capital accumulated over the life course were associated with FSS at age 43, for both women and men [47].	Cumulative poor social capital over the life was related to later mental ill-health.	
	Inequalities in Functional Somatic Symptoms (FSS) increased between the socio-economic groups from the age of 16 to 43. The gap was explained by social and material adversities during life [49].	Adversities explained the increasing socio-economic gradient in mental health during life.	***Relation between adversities, and the socio-economic gradient of mental ill-health during life***
	Socio-economic gradient in somatic health from the age of 16 to 30 was explained by poor social relationship (poor relationship with father and having unemployed friends among men, experiencing violence among women), economic hardship (financial strain among women) and poor health behaviors (high alcohol consumption among men and smoking among women) [50].		
	Adverse socio-economic status of the family at the age of 16 was associated with unfavorable material and social living conditions at the age of 21 and 30 which in turn was related to FSS at the age of 43 in men [51]		
	Experience of social adversities at the age of 16 was associated with entering an unfavorable trajectory of internalized mental health symptoms (IMHS) from the age of 16 to 43 both in men and women [52].	Early adversities were related to poor development of mental health during life.	
	Experience of social adversities at the age of 16 was associated with lower self- related health at the age of 43 in both men and women [53].	Early adversities were related to later mental ill-health.	
	Cumulative social and material adversities from the age of 16 to 43 was associated with experience of FSS in midlife (age 43) in both men and women independent of experience of FSS at the baseline [34].	Cumulative adversities over the life were related to later mental ill-health.	
	Perceived gender inequality in couple’s relationship and domestic sphere was associated with increased psychological distress in both couples [54], depressive symptoms among women and sub-optimal self-related health among men at the age of 43 [55, 56].	Gender equality in adult life was related to mental health.	***Relation between gender relations and mental health in adult life***
	Non-traditional gender ideology (supporting or practicing gender equality) at age 30 was associated with decreased risk of anxious symptoms in women. For men, non-traditional childcare at age 43 was associated with decreased risk of depressive symptoms [57]		
	Women’s responsibility for performance of housework increased from ages 30 to 43 but not men. These changes were associated with elevated levels of FSS at age 43 in women [58].		
	Inequality in housework, in combination with experiencing the couple’s relationship as gender-unequal, were associated with increased psychological distress in both men and women at the age of 43 [59].		
	Gendered division of housework was associated with increased experience of stress among men and women at the age of 47 [30].		

### Levels and themes

#### Macro-level

Macro-level usually referred to as the social “blueprint” and consists of broader societal economic, culture, values and politics. Social structures shaped at the macro-level could have cascading effect though out the interactions of all other levels and ultimately permeate micro-level of ever day life [62]. We identified two structures – class structures and gender order- that permeate all other levels of the Bronfenbrenner model. Class structures reflect a common situation on the labour market, in relation to resources and prerequisites for labour market carrier. Class is often measured by working-class-belonging which influence one’s access to social and material resources [63–65]. Gender order can be defined as the structure of gender relations in a given society at a given time and as it has been suggested by Connell, gender relations should be understood” as multidimensional: embracing at the same time economic relations, power relations, affective relations and symbolic relations; and operating simultaneously at intrapersonal, interpersonal, institutional and society-wide levels” [66].

#### Exo-level

##### Relations between neighbourhood disadvantage and mental ill-health during life

Exo-level refers to settings and social structures in which the individual is not an active participant but can influence him/her nonetheless [6, 7]. At the exo-level, several studies evaluated the importance of neighbourhood characteristics on mental health of the individuals. We found cumulative neighbourhood disadvantages over life to be related to later mental ill-health. Only among women, cumulative neighbourhood disadvantages from the age of 16 to 43 was significantly associated with FSS at the age of 43. The association was largely explained by parallel accumulation of social and material adversities at the individual-level [34]. Among men, cumulative neighbourhood disadvantages between 16 and 43 was associated with higher allostatic load at the age of 43, while no such association was observed among women [35]. A further study indicated that neighbourhood of living across the life-course explained 8% of variation in FSS at the age of 43. The variation was partly explained by neighbourhood of residence in adolescence [36].

Another study regarding exo-level exposure, showed that positive attitudes towards drunkenness among classmates was related to later alcohol consumption. Individuals who had several classmates with positive attitude towards heavy alcohol consumption at the age of 16 were more likely to get engaged in heavy episodic drinking at the age of 43 [37]. Classroom in our study can be representative of the neighbourhood as during 1980s in Luleå, individuals were selected to schools based on their living areas.

#### Meso-level

##### Relation between parental interaction with other settings and mental health during life

Meso-level refers to interaction between two or more micro-level settings in which the individual is an active participant [6, 7]. At this level, the importance of interaction between home and school, which was evaluated by measuring parental involvement in their offspring’s studies at the age of 16, was highlighted. Good parental interaction with school in early life, was related to mental health during life and later adulthood. Evaluating trajectories of IMHS indicated that both teacher-rated parental involvement in their offspring’s studies and student-rated availability of assistance with homework during the last year of compulsory school predicted development of more favourable trajectories of IMHS from the age of 16 to 43 years [38]. Further, parental interest in their offspring’s studies during the last year of compulsory education was associated with lower allostatic load at the age of 43. Academic achievements over life mediated the large part of this association [39]. Another example of parental interaction with other settings was raised in a qualitative study conducted on a subsample of early unemployed young women who were repeatedly interviewed between the age of 16 to 33. Th study showed parental interaction with destructive relationship in early life was related to mental health in early adulthood. One of the participants in the study narrated how her parents interacted with her abusive boyfriend and supported her to terminate the relationship [40].

#### Micro-level

##### Relation between poor social relationships and mental ill-health during life

Micro-level refers to the most proximal setting to the individual with which the individual interacts such as home, school, etc. [6, 7]. The importance of micro-level settings for mental health of the individuals were highlighted in several papers. The relation between poor social relationships and mental health of individuals during life was a recurrent theme in the studies. Poor relationship with family in early life was related to later mental ill-health symptoms and alcohol misuse. Individuals who had poor relationship with their parents at the age of 16 were more likely to have IMHS at the age of 30 and FSS both at 30 and 43 years of age [41]. Among men, poor relationship with family in adolescence was associated with episodes of heavy alcoholic drinking in early adulthood (at 21 and 30) [42].

Similar to relationship with family, negative effects of poor relationship with peers and classmates in early life on later mental health and alcohol misuse was observed among the participants. Poor relationship with peers at the age of 16 was associated with several indicators of ill mental health such as depressive symptoms, IMHS and FSS at the age of 43 [41, 43]. Among women, poor relationship with classmates at 16 were associated with increased likelihood of heavy episodic drinking at age 30 [42].

On the other hand, participant who had good school connectedness (defined as sense of belonging or psychological membership in the school or classroom) at the age of 16 showed lower levels of depressive and anxiety symptoms in midlife. The association appeared to be partially mediated by social and professional establishment in early adulthood. i.e., good school connectedness was associated with being in relationship or professionally active at the age of 30, which in turn were negatively associated with symptoms of depression and anxiety at the age of 43 [44].

Additionally, both social support and social capital were recognized as important determinants of mental health. Poor social support in early adulthood was related to current and later mental ill-health. Availability of structural (social integration) and functional (social attachment) support at the age of 30 were associated with a decrease in depressive symptoms in early adulthood (age30) and in mid-life (age 43) [45] whereas participants who had poor social support at the age of 30 were more likely to have IMHS at the age of 43 [46].

Similarly, social capital in early life was related to later mental health particularly among men. Adolescent boys with low social capital had higher level of FSS at the age of 43 [47] while civic engagement as an indicator of structural social capital, during adolescence was associated with a decrease in depressive symptoms in early adulthood among men [48]. In addition, cumulative poor social capital over the life was related to later mental ill-health. Low levels of social capital accumulated over life were associated with higher level of FSS in mid-life for both man and women [47].

#### Relation between adversities, and the socio-economic gradient of mental ill-health during life

Our results indicated that adversities explained the increase in socio-economic gradient in mental health during life. Social adversities included parental separation, loss or illness of parent or someone close, residential instability, participant’s own separation as well as threat or experience of violence. While material adversities were mostly related to physical living conditions such as parental poor standards of living and residential crowding.

Evaluating Functional Somatic Symptoms (FSS) from the age of 16 to 43 showed that the gap between socio-economic groups (with lower and higher socio-economic status) was small in early life but numerically increased with age. The inequalities in FSS were attributed to social and material adversities mainly during adolescence and moderately in midlife (age 30 and 43) [49].

Exploring the mechanisms behind the socio-economic gradient in symptoms of mental health indicated that poor social relationship (poor relationship with father and having unemployed friends among men, experience of violence among women), economic hardship (financial strain among women) and poor health behaviours (high alcohol consumption among men and smoking among women) could explain why men and women within lower socio-economic group had more symptoms of mental health than others [50].

Long-term effect of adverse family socio-economic status on mental ill-health was observed among the study participants. Among men, parental low socio-economic status during adolescence was associated with FSS at the age of 43. Assessing the possible pathways, indicated that among men, the association was partially explained by their own occupational class at the age of 21 and social and material adversities at the age of 30. No such association was evident for women [51].

Further, experience of social and material adversities during life were associated with symptoms of ill mental health in our studies. Early adversities were related poor development of mental health during life. Individuals who experienced social adversities at the age of 16 had higher risk of entering unfavourable developmental trajectories of internalized mental health over life. The influence of adversities was more pronounced at initial period of trajectories (at the age of 16) and attenuated over time [52]. In another study, experience of social and material adversities at the age of 16 was associated with lower self-related health at the age of 43. However, having advantaged situation with regards to school (such as higher average school marks, better general performance at school), peers (such as popularity among peers) and spare time (such as having quality spare time) at the age of 16 had protective effect on the detrimental effects of social and material adversities on participants later mental health [53]. Finally, cumulative adversities over the life were related to later mental ill-health. For both men and women, accumulation of social and material adversities from the age of 16 to 43 was associated with FSS at the age of 43 [34].

#### Relation between gender relations and mental health in adult life

The impact of gender equality in couple’s relationship on mental health of individuals was highlighted in several studies. Perceived gender inequality in couple’s relationship and domestic sphere at the age of 43 was associated with higher level of psychological distress in both men and women [54], depressive symptoms among women and sub-optimal self-related health among men [55, 56]. Women with non-traditional gender ideology (supporting or practicing gender equality) at age 30 showed lower anxious symptoms at the age of 43, while men who practiced non-traditional childcare (having similar/most/all childcare responsibilities compared to their partner) had lower level of depressive symptoms at the age of 43 [57]. Further, studies on the distribution of housework indicated that housework was highly gendered with women reporting higher responsibilities compared to the men. Such unequal distribution was associated with ill mental health symptoms in both men and women [30, 58, 59]. One example was the study measuring changes in housework over the course of adulthood, the study indicated that women’s responsibilities for performing the house works increased from the age of 30 to 43. This increase in amount and time of housework was associated with FSS among women [58]. Another study on the sample of men (*n* = 352) and women (*n* = 371) who were living with children at the age of 43 indicated that gender inequality in housework in combination with experiencing the couple’s relationship as unequal was associated with increased psychological distress both in men and women [59]. A qualitative study in a subsample of participants at the age of 47 indicated that for both men and women stereotypical gender practices in the housework was related to higher stress and deteriorated their wellbeing [30].

## Discussion

The aim of current integrative review was to identify social determinants outside paid work that are related to development of mental health over life in a 27-year prospective Swedish Cohort study by using Bronfenbrenner’s Ecological Systems Theory. To our best of knowledge this is the first synthesis of evidence regarding the determinants of mental health over life within such a framework. Synthesis of quantitative and qualitative studies revealed that class structures and gender order at the macro-level permeate the other levels and embodied as mental ill-health at the individual-level during life.

Class structure refers to hierarchical ranking of the people in the society based on their employment relations in the labour market. Such categorization indicates uneven distribution of power and status between classes and captures inequality within society. Social class determines one’s access to income, wealth, education, occupation and area of residence as well as exposure to life stressors and adversities [67]. There is mounting evidence that individuals with lower social class are at higher risk of physical and mental ill-health and the impact of low social class on health and wellbeing of individuals can accumulate over time [68].

Class structures permeate the neighbourhood of residence at the exo-level, expressed in terms of social disadvantages which in turn can cause mental health problem among inhabitants. We found early and cumulative neighbourhood disadvantages to be related to later ill mental health. The link between neighbourhood disadvantages and depression, anxiety and suicidal thoughts above and beyond individual attributors has been reported in previous studies [69–71]. The association between neighbourhood disadvantages and poor mental health might be related to cumulative exposure to stressors such as socio-economic problems, lack of resources and interpersonal violence without sufficient protective social resources to buffer their effects [72].

At the exo-level, we also found early exposure to positive attitudes towards drunkenness at class level was related to later alcohol consumption among participants. According to social learning theory, the general culture and structure of communities such as classroom or school might create a learning environment where the norms and behaviours are shaped [73]. Studies on the long-term effects of exposure to classroom attitude toward drunkenness are rare. However, in a study conducted among students in 4 and 5th grade in US, prevalence of alcohol and substance consumption at the classroom level (the reported use among all other pupils in the class) was a major predictor of substance use among students [74].

Similar to the previous level, class structures permeate the meso-level and influence parental interactions with other settings such as schools. Working-class parents have limited educational, social, cultural and economic resources important for facilitating their interaction with school which in turn can affect mental wellbeing of their children [75, 76]. We found parental interaction with other settings such as school to be positively related to mental health during life among participants. Comparable to our findings, higher parental involvement in academic development of their children was associated with better social skills and emotional functioning as well as fewer behaviour problems among children in other studies [77, 78].

At the micro-level the importance of social relationships such as relation with family and peers as well as social support and social capital was brought up in several studies. Class structure can permeate into micro-level interactions and influence social relationships and ultimately affect mental health of individuals over their life. Financial strain, living in disadvantaged neighbourhood and limited access to recourses among working-class parents might affect their social relationships as well as their relationship with their children. For example, working-class parents will, due to financial problems, have a more stressful life which in turn might diminish their time and other possibilities of creating good social relationships. Ultimately these problems can affect the mental wellbeing of their children [79]. In this review, poor relationship with family in early life was related to later alcohol misuse and ill mental health among participants. The bond between impaired family relationships and poor mental health in adult life has been suggested in several studies [80, 81]. Good relationship with family can enhance the sense of security and coherence among individuals and has been shown to be associated with sense of identity, social competence, emotional adjustment and self-esteem [82, 83].

Beside family relationship, poor relationship with peers during adolescence was related to poor mental health and alcohol consumption in later life in our study. Lack of financial recourses and higher level of stress among families with working-class belong, might reduce self-esteem in the children and negatively affect the quality of their relationship with their peers [84]. Quality relationship with peers particularly during adolescence has been suggested as an important element for social and emotional growth of individuals. Such relationship can evolve sense of identity and connectiveness among adolescents and result in better mental health during life [85]. In line with findings of this review, causal relationship between poor peer relationship in early life and depression in later life has been reported in previous studies [86, 87].

Social support and social capital were other important determinants of mental health in our sample. Class structure can affect the size and composition of “social network” among those with working-class belonging and reduce their ability in mobilizing information and expertise necessary for dealing with difficulties [88]. Both short term and long-term effects of social support on mental health of participants were detected in our study. A great body of evidence exists about positive association between social support and mental health and wellbeing of individuals [89]. Social support can influence one’s mental health trough sustaining sense of mattering, self-esteem, control, belonging and companionship as well as through perceived support availability [90]. Similarly, long-term and cumulative effects of social capital on mental health of individuals were observed in our study. The link between social capital and mental health has been reported in several studies [91, 92]. Social capital might influence individual’s mental wellbeing by providing feeling of stability, belonging, access to resources and also by widening the circle of trust and enabling social mobility [93, 94].

Class structures at the macro-level can permeate micro-levels and create early and cumulative disadvantages (such as lower socio-economic status and social and material adversities) among those with working-class belonging and ultimately embody as mental ill-health during life. In our review both parental and individual’s own socio-economic status was related to inequality in mental health during life and individuals who had lower socio-economic status were more likely to experience mental health symptoms, a finding that is consistent with previous research [95, 96]. We found experience of both early and cumulative adversities during life to be related to poor mental health. In line with our findings, in a systematic review of 37 studies, adverse childhood experiences were moderately and strongly associated with heavy alcohol consumption and ill mental health respectively in later life [97]. Further the association between experience of cumulative adversities and poor mental health during life has been reported in other studies [98, 99].

Gender order refers to historically constructed patterns of power relations between men and women in a given society. The gender order defines the construction of femininities and masculinities which are discursively produced within a certain society [100]. Similar to class structure, gender order at the macro-level of the society can permeate into other levels and eventually embody in the individuals as mental ill-health. Women in our study had higher level of internalized mental health symptoms while alcohol misuse was higher among men, a finding that has been constantly reported in other studies [101, 102]. Further in line with previous studies, we found that mental health effects of social and material stressors can be different among men and women. This might be due to gender relations and their interplay with other determinants of mental health [101, 103].

Gender order at the macro-level can also permeate into the micro-level of couple’s relationship and become embodied as mental health symptoms. In our studies, consistent with other studies, women disproportionally were responsible for most of the housework [104, 105]. We found perceived gender inequality in the couple’s relationship and division of housework to cause mental illness. The association between perceived inequality in domestic sphere and psychological ill-health particularly among women has been reported in several studies [106–108]. Perceived gender inequality might negatively affect the sense of control [109] and create feeling of unfairness [110] and relationship unsatisfaction [111] and result in lower mental health among couples.

### On the method

The strength of this review was the longitudinal approach by including studies from a 27-year follow-up of a cohort of school leavers. The cohort by itself had extremely low attrition rate with repeated follow ups over a long period. Using Bronfenbrenner socio-ecological model allowed for identification of mental health determinants at multiple levels, while including also qualitative studies enabled us to present more in-depth view on the determinants. The measurements of mental health have been validated against current golden standards of psychiatric research and have been shown to have acceptable factorial invariance and internal consistency over time [29]. The issue of generalizability needs to be addressed. Although the cohort showed to be comparable with the total Swedish population with regards to socio-demographic, socio-economic, health status and health behaviour [28], due to the closed nature of the cohort the sample were more homogeneous compared to the Swedish population of today. Further the study has been done in a high-income country which makes it difficult to generalize the results to the low- and middle-income settings. However, several determinants of mental health which were brought up in this review have been shown to be also relevant in low- and middle-income countries [112]. Lastly, in this review limited studies evaluated factors related to mental health of individuals at the meso-level. According to Bronfenbrenner, strong meso-level interactions are essential for successful development and mental health of individuals [6, 7], thus further research focusing on determents of mental health at this level is warranted.

## Conclusions

This review has shed light on how social structures at macro-level, namely class structures and gender order can permeate into other levels and finally become embodied in the individuals as symptoms of mental ill-health over life. As it has been suggested by Marmot, interventions aiming to close the (mental) health gap between men and women as well as between social groups should try to improve the gendered and classed conditions of daily life i.e., the circumstances in which the people are born, grow, live, work and age; efforts should be made to reduce gender and class-related inequalities in distribution of resources and public awareness should be raised about social determinants of (mental) health [96] . Further, findings derived from this review indicated that mental health of individuals at each stage of life is the result of both unique and common factors manifesting at different socio-ecological levels. As the interplay and effects of these factors changed during life, interventions and policies must be appropriate for each stage of life and at different levels. Additionally, we have found that several determinants of mental health during adolescence have long-term effect on mental wellbeing of the individuals during life. Adolescents has been suggested as the onset period for most of the mental health disorders as well as the time of greatest change and diversity in exposure to social determinants of mental health [113]. Thus, interventions targeting adolescent mental health by reducing gaps between social groups, addressing neighbourhood disadvantage, improving parental interaction with both their children and their surrounding settings, improving social relationships might be of especial importance for promoting individuals’ wellbeing over their life.

## Data Availability

The data is not publicly available because the Swedish Data Protection Act (1998:204) does not permit sensitive data on humans (like in our study) to be freely shared. The datasets are available based on ethical permission from the Regional Ethical board in Umeå, Sweden, from one of the co-authors (Anne Hammarström).

## Abbreviations

FSS: Functional Somatic Symptoms
HED: Heavy Episodic Drinking
IMHS: Internalized Mental Health Symptoms
NoSCo: Northern Swedish Cohort
WHO: World Health Organization
